# *Aster glehni* F. Schmidt Extract Modulates the Activities of HMG-CoA Reductase and Fatty Acid Synthase

**DOI:** 10.3390/plants10112287

**Published:** 2021-10-25

**Authors:** Hyunbeom Lee, Hyoung Ja Kim, Hyungi Chae, Na Eun Yoon, Byung Hwa Jung

**Affiliations:** 1 Molecular Recognition Research Center, Korea Institute of Science and Technology, Seoul 02792, Korea; hyunbeom@kist.re.kr (H.L.); khj@kist.re.kr (H.J.K.); cogusrlchg@naver.com (H.C.); betteryun@kist.re.kr (N.E.Y.); 2 Department of HY-KIST Bio-Convergence, Hanyang University, Seoul 04763, Korea; 3 Division of Bio-Medical Science & Technology, KIST School, Korea University of Science and Technology, Seoul 02792, Korea

**Keywords:** *Aster glehni* F. Schmidt, di-caffeoylquinic acid, fatty acid synthase, HMG-CoA reductase, LC–MS/MS, polyphenols

## Abstract

*Aster glehni* F. Schmidt (AG), is a natural product known to have anti-obesity effects, but the mechanism underlying these effects is not well documented. We hypothesized that AG may have inhibitory effects on enzymes related to lipid accumulation. Herein, AG fractions were tested against HMG-CoA reductase (HMGR) and fatty acid synthase (FAS), two important enzymes involved in cholesterol and fatty acid synthesis, respectively. We found that dicaffeoylquinic acid (DCQA) methyl esters present in AG are largely responsible for the inhibition of HMGR and FAS. Since free DCQA is a major form present in AG, we demonstrated that a simple methylation of the AG extract could increase the overall inhibitory effects against those enzymes. Through this simple process, we were able to increase the inhibitory effect by 150%. We believe that our processed AG effectively modulates the HMGR and FAS activities, providing promising therapeutic potential for cholesterol- and lipid-lowering effects.

## 1. Introduction

Dietary supplements and herbal medicines derived from natural products purified or isolated from edible plants have always been popular due to their effectiveness and lower toxicity [1,2]. Approximately 50%–80% of the Asian and African population reportedly takes herbal medicines according to the World Health Organization (WHO) [3]. Due to their demonstrated safety and effectiveness, drugs derived from natural products comprise approximately a quarter of the total drug market [4]. Efforts to develop new drugs based on plant extracts or their derivatives are ongoing [5]. In this study, we were interested in the native, wild plant *Aster glehni* F. Schmidt (AG), a natural product mostly found on Ulleung Island, Korea. The young stems and leaves of AG collected in the spring are often consumed by Koreans as a common vegetable side dish, Chwinamul [6]. Recently, Lee et al. reported that AG extract may improve NAFLD conditions through regulating PPAR δ and adiponectin [7]. Additionally, it has been reported that AG has various therapeutic effects, such as anticonvulsant, antioxidant, anti-inflammatory, hypouricemic, and anti-obesity effects [6,8,9,10].

Cardiovascular diseases (CVDs) are the leading cause of death worldwide according to the World Health Organization [11]. Strokes and heart attacks, classified as blood vessel CVDs, are mainly caused by a blockage, such as a build-up of fatty deposits on the inner walls of blood vessels, which prevents blood from flowing to the brain or heart [12]. Fatty deposits such as cholesterol and saturated fatty acids are biosynthesized through the mevalonate pathway and fatty acid synthesis pathway, respectively [13,14]. One way to lower blood cholesterol levels is by inhibiting 3-hydroxy-3-methylglutaryl-coenzyme A reductase (HMGR) in the mevalonate pathway. This pathway catalyzes the conversion of HMG-CoA to mevalonic acid, a precursor molecule of cholesterol. Statins are well known for lowering plasma cholesterol levels by inhibiting HMGR, and their therapeutic potential for treating patients with coronary artery disease and heart diseases has been demonstrated [15]. Recently, De Silva et al. reported that elevated levels of circulating serum fatty acids were observed in patients with cerebrovascular disease [16]. The biosynthesis of saturated fatty acids is modulated by fatty acid synthase (FAS) from acetyl-CoA and malonyl-CoA. Inhibitors of FAS, such as cerulenin, have shown to be effective against obesity and related diseases [17,18,19]. As we believe that HMGR and FAS are the two enzymes critical for regulating cholesterol and fatty acid levels, we hypothesized that AG might show its anti-obesity effects through modulating the activities of these enzymes.

**Figure 1:**
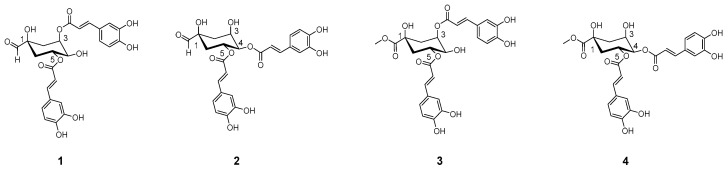
Components known to be present in AGE: 3,5-di-o-caffeoylquinic acid (**1**), 4,5-dicaffeoylquinic acid (**2)**, 3,5-dicaffeoylquinic acid methyl ester (**3**) and 4,5-dicaffeoylquinic acid methyl ester (**4)**.

During our search for biologically active components from the natural product, we found that the ethyl acetate fraction prepared from *Aster glehni* (AGE) showed an inhibitory effect on HMGR and FAS. Previously, dicaffeoylquinic acids (DCQA) **1**–**4** were identified in the AGE (Figure 1) [6], and among them, 3,5-dicaffeoylquinic acid (1) and 4,5-di-o-caffeoylquinic acid (**2**) were found to be the major components of the AGE. DCQAs were recently reported for their anti-inflammatory and antioxidant activities [20]. To extend our knowledge of the anti-obesity effects of AG, we focused on the DCQA series for their activities related to HMGR and FAS inhibition. In this way, we have identified that the methylated DCQA **3** and **4** have better inhibition toward HMGR and FAS. Here, we demonstrate that general esterification of the AGE to methylated AGE (AGEM) increases its potency against HMGR inhibition, and through sequential fractionation of AGEM, we report the components present in the most potent fractions responsible for inhibiting both enzymes.

## 2. Results

### 2.1. HMG-CoA Reductase Inhibition Assay

The crude mixture of AG was extracted sequentially with methanol, dichloromethane, ethyl acetate, and butanol. Among the extracted fractions, the ethyl acetate fraction (AGE) had the most antioxidants and HMGR inhibition activities (data not shown). The most abundant compound found in the AGE was 3,5-dicaffeoylquinic acid (**1**) [6]. Among the series of DCQA compounds, 3,5-dicaffeoylquinic acid methyl ester (**3**) showed the most potent activity against HMGR inhibition with an IC_50_ value of 9 μM. An analog of (**2**), 4,5-dicaffeoylquinic acid methyl ester (**4**) also showed a reasonable inhibitory activity of 63 μM (Figure S1).

### 2.2. Esterification of AGE

**Figure 2:**
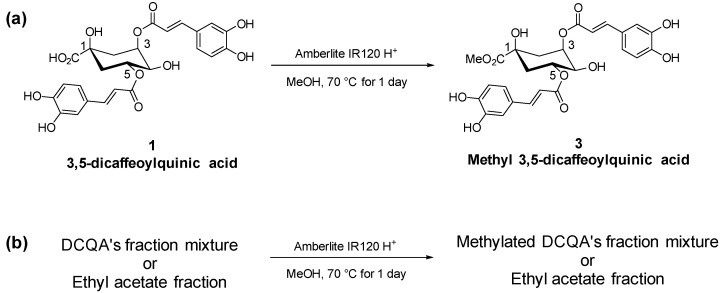
Scheme for esterification of (**a**) 3,5-DCQA into methyl 3,5-DCQA and (**b**) AGE mixture into methyl esters of AGE mixture were performed using Amberlite IR 120 H^+^.

As shown in Figure 2, compound **1** was successfully methylated to produce compound **3** using Amberlite IR120 H^+^ and methanol. Esterification was performed on the AGE using the same method to produce the methylated mixture AGEM.

### 2.3. Concentration Dependent HMG-CoA Reductase Inhibition Assay

**Figure 3:**
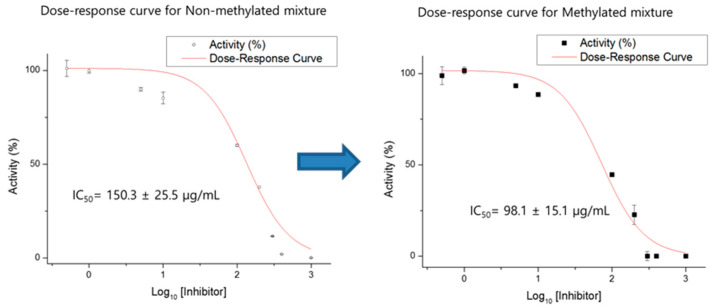
Dose–response activity curve for non-methylated (**left**) fraction and methylated (**right**) fraction against HMGR. After simple methylation, the IC_50_ values increased from 150.3 ± 25.5 µg/mL to 98.1 ± 15.1 µg/mL.

A concentration-dependent HMGR inhibition study shows the IC_50_ values of the mixture. After the esterification of the extract to increase the methylated DCQA contents in the mixture, the methylated ethyl acetate fraction of AG and AGEM showed better HMGR inhibitory effects, as shown in Figure 3. The AGEM had an IC_50_ value of 98±15 μg/mL, while the AGE originally had an IC_50_ value of 150 ± 25 μg/mL. A simple esterification of the AGE increased its potency by approximately 150%.

**Figure 4:**
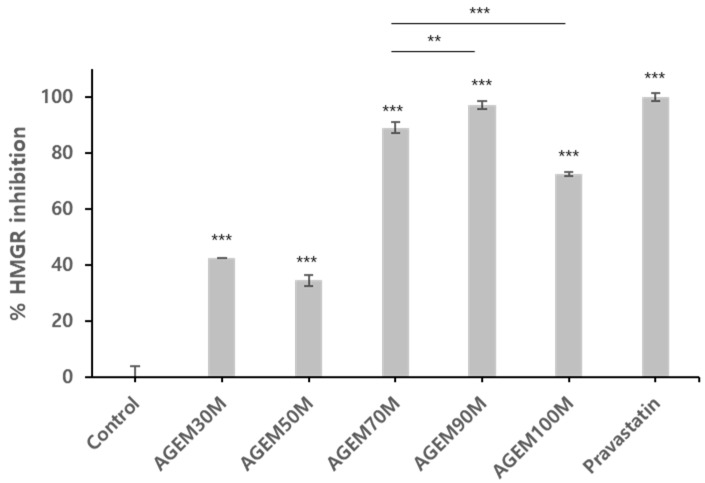
HMGR inhibition assay with AGEM30M through AGEM100M at 100 g/mL. AGEM70M and AGEM90M showed best inhibitory activity against HMGR. Pravastatin, 10 μM (positive control), showed good inhibition against HMGR. Statistical significance was analyzed using Student’s *t*-test *** *p* < 0.001, ** *p* < 0.01. Asterisks above the bar represents the statistical significance comparing each group (*n* = 3) to the control group.

To identify the most active portion of the AGEM, it was fractionated again and tested for activity against HMGR. As shown in Figure 4, the fractions AGEM70M, AGEM90M, and AGEM100M showed better potency against HMGR inhibition, while AGEM30M and AGEM50M had less activity. The most active fractions, AGEM70M and AGEM90M, were tested for their concentration-dependent enzymatic activity, and it was found that they had IC_50_ values of 19.7±1.4 μg/mL and 18.6±0.85 μg/mL, respectively, for HMGR inhibition (Figure S2).

### 2.4. Concentration-Dependent Fatty Acid Synthase Inhibition Assay

**Figure 5:**
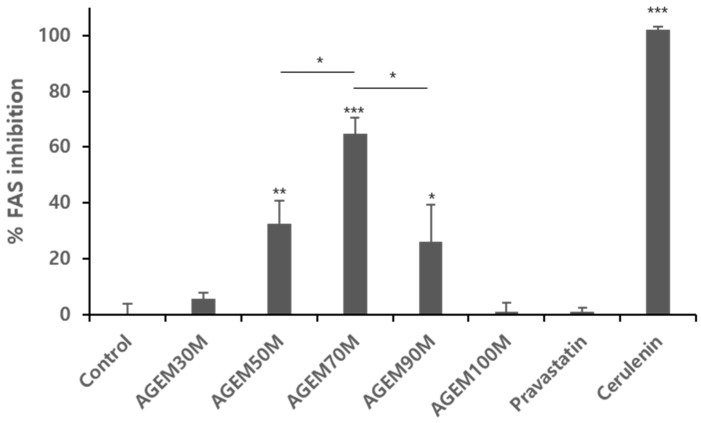
FAS inhibition assay with AGEM30M through AGEM100M at 100 g/mL. AGEM70M showed the most inhibition against FAS. Cerulenin (positive control), an inhibitor of FAS, showed good inhibition against FAS, while pravastatin showed no effect against FAS. Statistical significance was analyzed using Student’s *t*-test *** *p* < 0.001, ** *p* < 0.01, * *p* < 0.05. Asterisks above the bar represents the statistical significance comparing each group (*n* = 3) to the control group.

As shown in Figure 5, among the AGEM fractions, AGEM70M showed the best inhibition activity against FAS. A concentration-dependent inhibition study showed that AGEM70M has an IC_50_ value of 106.1 μg/mL (Figure S3). Interestingly, AGEM90M and AGEM100M, which both showed good HMGR inhibition activity, showed only weak FAS inhibitory activity.

### 2.5. Qualitative Analysis of AGEM and Quantitative Analysis of AGEM70M

**Figure 6:**
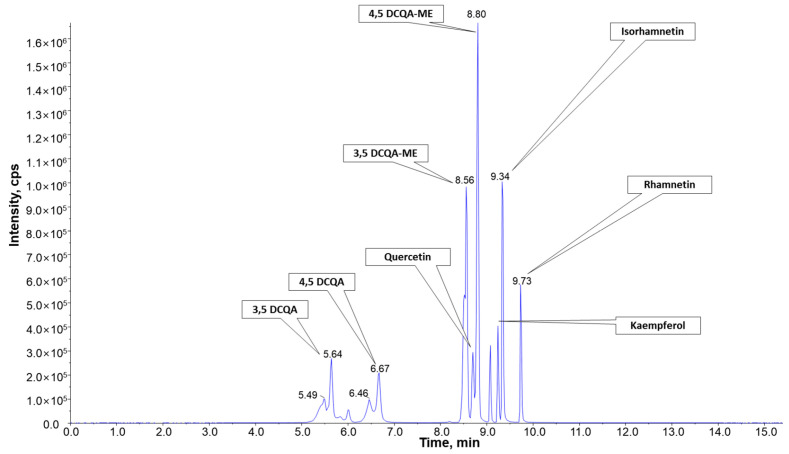
Chromatogram of the components in AGEM70M fractions from LC–MS/MS elution. The components eluted from the column are identified as 3,5-dicaffeoylquinic acid (3,5-DCQA), 4,5-dicaffeoylquinic acid (4,5-DCQA), quercetin, 3,5-dicaffeoylquinic acid methyl ester (3,5-DCQA-ME), 4,5-dicaffeoylquinic acid methyl ester (4,5-DCQA-ME), kaempferol, isorhamnetin, and rhamnetin.

AGEM fractions were qualitatively analyzed using LC–MS/MS. Various compounds, including a DCQA series, a glucopyranoside series, and polyphenols, were detected from the AGEM fractions (Figure S4). As AGEM70M showed the most inhibition against HMGR and FAS, the components present in AGEM70M were analyzed qualitatively using LC–MS/MS, as shown in Figure 6. Each fraction was analyzed, and the components were quantified using standard compounds, and these results are summarized in Table S1.

## 3. Discussion

Today, herbal medicines are widely used to treat medical illnesses or as dietary supplements due to their various therapeutic potentials. Particularly for treating hyperlipidemia, a risk factor of CVD, the tendency to use synthetic drugs is decreasing due to the unwanted side effects [21,22,23]. Accordingly, the use of herbal medicines with lipid-lowering potentials to complement or to replace synthetic drugs is increasing [24].

Although AG has been shown to be effective for antiadipogenic and anti-obesity activities, its exact lipid-lowering mechanism remains unknown [8]. As the plant extracts may contain multiple components that may interact and interfere mechanistically, poly-pharmacological effects are known as multi-target effects [25,26]. We hypothesized that the active compounds in AG may modulate the enzymes involved in lipid accumulation. We targeted two of the important enzymes involved in cholesterol and lipid biosynthesis, HMGR and FAS.

First, we identified 3,5-dicaffeoylquinic acid methyl ester (**3**) as the most active compound, which is isolated from AG for inhibiting HMGR. As 3,5-dicaffeoylquinic acid (**1**) is the most abundant compound present in the AGE fraction, we attempted a general esterification of the AGE mixture to increase the volume of the 3,5-dicaffeoylquinic acid methyl ester (**3**). We hypothesized that the HMGR inhibitory activity would generally increase through simple esterification of the AGE mixture. As anticipated, the activity against HMGR inhibition significantly increased through simple esterification of AGE into AGEM (Figure 3). Surprisingly, AGEM also showed inhibitory activity against FAS (data not shown).

FAS is an enzyme that catalyzes the formation of palmitic acid from malonyl-CoA and acetyl-CoA. The modulation of FAS activity is implicated in various diseases, including cardiovascular diseases and cancers. Additionally, FAS is known to be overexpressed in various cancer cells, such as breast and prostate cancer cells, and has been well documented previously as a potential therapeutic target [27,28,29]. Thus, the AGEM was fractionated, and the fractions were tested for their inhibitory activity against HMGR and FAS.

As shown in Figure 4 and Figure 5, among the AGEM fractions, AGEM70M showed good inhibition activity against HMGR and FAS. However, AGEM90M and AGEM100M, which both showed good HMGR inhibition activity, showed only weak FAS inhibitory activity. Cerulenin, a known FAS inhibitor [17], showed very good potency against FAS, while pravastatin, a potent inhibitor of HMGR, did not have any effect on FAS, confirming that the mode of action for HMGR and FAS inhibition differs. This suggests that there may be more than one active compound present in the AGEM70M fraction and the active compounds that contribute to inhibiting each enzyme are different.

As AGEM70M is a dual-modal active fraction that inhibits both HMGR and FAS, we performed qualitative and quantitative analyses of the fractions to identify the active components that are responsible for these dual effects. As shown in Figure 6, DCQA series and multiple polyphenolic contents, such as kaempferol, rhamnetin, isorhamnetin, and quercetin, were identified in the AGEM70M fraction. In multiple studies, plant polyphenols were shown to be effective in lowering cholesterol and lipid levels by modulating HMGR and FAS activities [30,31,32,33]. Thus, not only the active DCQA contents in AGEM70 but also the polyphenols may have been responsible for the dual-modal activity.

Since AGEM70M had activities against both HMGR and FAS, we further fractionated it into six fractions (AGEM70M-1 to AGEM70M-6) to analyze the active components responsible for the activity against each enzyme. As shown in Figure S5, all fractions had the potency to inhibit HMGR, but the level of inhibition differed from AGEM70M-1 to AGEM70M-6.

Among the fractions, AGEM70M-2 and -3 showed reasonable HMGR inhibitory activity, which may be attributable to the high content of methylated DCQA, **3** and **4**. However, the highest level of HMGR inhibition was observed in the AGEM70M-4 fraction. Since we found that isorhamnetin and kaempferol also show weak HMGR inhibitory effects (Figure S6), we believe the high activity in AGEM70M-4 may be due to the synergistic effect of **3**, **4**, kaempferol, and isorhamnetin.

## 4. Materials and Methods

### 4.1. Reagents

All reagents and materials were purchased from Sigma-Aldrich (MO, USA), except the following: MCF-7 cells were purchased from the Korean Cell Line Bank (Seoul, Korea). Dulbecco’s Modified Eagle’s Medium (DMEM), antibiotic-antimycotic, fetal bovine serum, and phosphate-buffered saline were purchased from Gibco™ (Thermo-Fisher Scientific, Waltham, MA, USA).

### 4.2. Instruments

Enzyme assays were recorded on a Versamax microplate reader (Molecular Devices, San Jose, CA, USA) with transparent 96-well plates (Greiner Bio-One, Kremsmünster, Austria). Qualitative analyses for the components in the AG extracts were performed on an Ultimate 3000 UHPLC system with an auto-sampler and a column oven coupled to an LTQ Orbitrap Velos Pro™ system mass spectrometer (Thermo Scientific, Waltham, CA, USA) with a heated electrospray ionization source (HESI). The software packages Xcalibur2.2, TunePlus2.7, and Chromeleon MS Link 6.80 (Thermo Scientific, Waltham, CA, USA) were used. Quantitative analyses for the components in the AG extracts were performed on an ExionLC AD System connected to a Triple Quad™ 4500 system mass spectrometer equipped with an electrospray ionization source (AB Sciex, Redwood City, CA, USA).

### 4.3. Plant Materials

The AG leaves were collected from Ulleung Island, Korea, during August, 2007, and identified by prof. Chang-Soo Yook, Kyung Hee University. The voucher specimens (971-12A) were deposited in the herbarium of the Korea Institute of Science and Technology. The AG was extracted as previously reported [6].

### 4.4. HMG-CoA Reductase (HMGR) Inhibition Assay

The HMGR activity assay was optimized using a 96-well microplate reader to measure the amount of NADPH oxidation during the process of enzyme turnover. Each well contained 89 μL of 50 mM sodium phosphate buffer (pH 6.8), 0.8 mM NADPH and 2 μg of enzyme (2–8 units/mg); 1 μL of a desired concentration of an inhibitor sample in DMSO and 0.8 mM HMG-CoA in 10 μL of phosphate buffer was added to the well to start the reaction. As a positive control, 10 μM pravastatin was used, while blank DMSO was used as a negative control. The HMGR activity was determined by monitoring the decrease in NADPH absorbance at 340 nm using a 96-well microplate reader for 900 sec at 37 °C. All assays were performed in triplicate, and all data are presented as the mean ± standard deviation. Statistical analysis was performed using Student’s *t*-test, comparing each group to the control group. The results are represented by asterisks *** *p* < 0.001.

### 4.5. Esterification of the Ethyl Acetate Fractions of AG (AGE)

Chopped and air-dried aerial parts of AG (2.7 kg) were extracted three times with methanol (26 L) at room temperature to produce a methanol-soluble extract. The dried extract residue (394.8 g) was suspended in water and then partitioned in turn with dichloromethane, ethyl acetate and *n*-butanol. The ethyl acetate fraction (AGE) of 506 mg was refluxed with methanol (20 mL) and acidic resin (Amberlite IR 120 H^+^, 500 mg) for 1 day and then filtered to remove the resin. The methanol was evaporated under reduced pressure to yield 530.3 mg of residue (AGEM).

### 4.6. Fractionation of the Methylated AGE (AGEM)

AGEM was divided into five fractions by Diaion HP-20 using 30% (AGEM30M), 50% (AGEM50M), 70% (AGEM70M), 90% (AGEM90M), and 100% MeOH (AGEM100M) as the eluent. The AGEM70M (89 mg) fraction was purified by a Sephadex LH-20 CC using MeOH as the eluent to give six sub-fractions (AGEM70M-1-6), which were stored at −20 °C until required, at which point, each sub-fraction was thawed at room temperature and dissolved in DMSO (10 mg/mL) for a stock solution and used for assay.

### 4.7. Fatty Acid Synthase (FAS) Inhibition Assay

FAS activity assay was performed using a protocol as described previously [27]. Briefly, human breast adenocarcinoma MCF-7 cells were obtained from the Korean Cell Line Bank and maintained in DMEM containing 10% heat-inactivated fetal bovine serum, 100 units/mL penicillin, an antibiotic-antimycotic agent including 100 µg/mL streptomycin, and 0.25 µg/mL Fungizone^®^ (amphotericin B) in a humidified incubator at 37 °C containing 5% CO_2_. Confluent cells were harvested by trypsinization and subcultured. After harvesting with trypsin-EDTA, MCF-7 cells were lysed mechanically through three cycles of freeze-thawing by using 150 L of 0.2 mM phosphate buffer, pH 7.0, containing 125 μg/mL of a protease inhibitor cocktail. After centrifuging at 1,500×g for 10 min at 4 °C, the supernatant was collected and treated with 10% ammonium sulfate for precipitation. The sample was centrifuged again, and the supernatant was treated with 15% ammonium sulfate, which precipitates FAS. The collected FAS crude mixture dissolved in the buffer was then washed three times using a 100K MWCO Amicon Ultra-0.5 mL centrifugal filter (Merck Millipore, Burlington, MA, USA) at 14,000 rpm. The concentrated mixtures that remained in the centrifugal unit were used for the FAS activity assays. Fatty acid synthase activity was tested by measuring the amount of NADPH oxidation. All assays were performed in triplicate, and all data are presented as the mean ± standard deviation. Statistical analysis was performed using a Student’s *t*-test, comparing each group with the control group. The results are represented by asterisks *** *p* < 0.001, ** *p* < 0.01, * *p* < 0.05.

The activities for the overall FAS reaction were determined at 37 °C in 100 μL of assay buffer (0.2 M potassium phosphate, 1 mM DTT, 1 mM EDTA, pH 7.0, 1 μL inhibitor dissolved in DMSO) using 0.5 mM NADPH, 0.4 mM Malonyl-CoA, 0.24 mM Acetyl-CoA, 1% DMSO and 40 μL of a crude 2 mg/mL fatty acid synthase mixture. As a positive control, 100 μM of cerulenin was used, while DMSO was used as a negative control. The rate of the reaction was determined by monitoring the decrease in NADPH absorbance at 340 nm using a 96-well microplate reader.

### 4.8. Qualitative Analysis of Components in AGEM

The ACQUITY UPLC^®^ BEH C18 column (2.1 mm × 100 mm, 1.7 μm, Waters, MA, USA) was used for all chromatographic separations. The mobile phase was composed of 0.1% formic acid in 1% acetonitrile (*v/v*, mobile phase A) and 99% acetonitrile (*v/v*, mobile phase B). At a flow rate of 0.4 mL/min, the elution gradients were as follows: 0–1.5 min, 5 to 20% (B), 1.5–6.5 min, 20 to 25% (B), 6.5–10 min, 25 to 80% (B), 10–12 min, 80% (B), 12–13.5 min 80 to 5% (B), and 13.5–16 min 5% (B). The column was maintained at 40 °C, and the injection volume was 10 μL for each sample. The samples were kept at 4 °C in an autosampler during the analysis. The method for performing mass spectrometry using the LTQ Orbitrap Velos ProTM was optimized as follows: for the MS full scan mode, resolution, 60,000; scan range, 110–2000 m/z; and for the MS/MS mode, resolution, 17,500; AGC target, 2E04; scan range, relative to parent mass.

### 4.9. Quantitative Analysis of DCQA and Isoflavonoids in AGEM

The standards were serially diluted to acquire a calibration curve. The standards used were dicaffeoylquinic acid (DCQA)-methyl ester, DCQA, rhamnetin, isorhamnetin, kaempferol, quercetin, and quercetin-d_3_. AG fraction samples were diluted to 1 μg/mL with MeOH containing 0.5 μg/mL quercetin-d_3_ and readied for injection. The LC and parameters used for the analysis were as follows: ExionLC AD System (AB Sciex, CA, USA); ACQUITY UPLC BEH C18 Column (2.1 mm × 100 mm, 1.7 µm); injection volume, 5 μL; autosampler temp., 6°C, column temp., 40°C, mobile phase (A), 0.1% formic acid in 5% ACN; mobile phase (B), 0.1% formic acid in 95% ACN; flow rate, 0.4 mL/min; and running time, 16 mins. The gradient method was as follows: 0–0.5 min, 0 to 2% (B); 0.5–1 min, 2% (B); 1–1.5 min, 2 to 15% (B); 1.5–6.5 min, 15 to 17% (B); 6.5–10 min, 17 to 80% (B); 10–12 min, 80% (B); 12–12.5 min, 80 to 0% (B); and 12.5–16 min, 0% (B). The MS linked to the LC and the parameters for the analysis were as follows: Triple Quad 4500 system (AB sciex, US); ion source, turbo spray (ESI); polarity, positive; vaporizer temp, 500 °C; curtain gas, 35 psi; collision gas, 8 psi; ion spray voltage, 5500 V; nebulizer gas, 50 psi; turbo gas, 50 psi. The analyte MRM transition pair is shown in the following list.
Compound*m/z* (Q1, Q3)DCQA517.197163.027DCQA-ME531.177163.000Rhamnetin317.003273.900Isorhamnetin317.003302.000Kaempferol286.977153.000Quercetin302.950229.000Quercetin-d_3_306.058232.000

## 5. Conclusions

In summary, for the first time, we targeted two of the important enzymes, HMGR and FAS, involved in cholesterol and lipid biosynthesis that may be important in regulating lipid metabolism as therapeutic targets of AG. In this way, we identified 3,5-dicaffeoylquinic acid methyl ester (**3**) as the most active compound that is isolated from AG for inhibiting HMGR. As 3,5-dicaffeoylquinic acid (**1**) is the most abundant compound present in the AGE fraction, we performed a general esterification of the AGE mixture to generate a mixture, AGEM, with 150% increased activity. Furthermore, we found that AGEM70M is an active fraction that inhibits both the HMGR and FAS. Through qualitative and quantitative analyses, we identified the components present in the fraction, and through enzyme inhibition assays, we identified the active components that are responsible for these dual effects: **3**, **4**, kaempferol, and isorhamnetin. AG is a daily consumable food that is free from toxicity or unwanted side effects. Thus, we believe that processed AG extracts have therapeutic potential as a complementary and alternative medicine for lipid lowering agents.

## Data Availability

All data generated or analyzed during this study are included in this published article.

## Supplementary Materials

The following are available online at https://www.mdpi.com/article/10.3390/plants10112287/s1, Figure S1: Concentration dependent inhibition of HMGR by single DCQA components isolated from AG. Figure S2: A comparison of the dose–response activity curve for AGEM70M and AGEM90M. Figure S3: FAS inhibition assay of the fraction AGEM70M. Figure S4: LC–MS/MS chromatogram of AGEM fractions. Figure S5: HMGR inhibitory activity of the AGEM70M fractions. Figure S6: HMGR inhibition by polyphenols present in AGEM. Table S1: Quantitative analysis of the AGEM70M fractions.
