# Supplementary material for: Aster glehni F. Schmidt Extract Modulates the Activities of HMG-CoA Reductase and Fatty Acid Synthase
Source: Plants (Basel). 2021 Oct 25;10(11):2287. doi: 10.3390/plants10112287 (PMC8620592; doi:10.3390/plants10112287)
Supplement: Supplementary file 1 [file plants-10-02287-s001.zip › plants-1405498-supplementary.pdf]

<Supplementary Materials>

Aster glehni F. Schmidt extract modulates the activities of HMG-CoA reductase and fatty acid synthase.

Hyunbeom Lee[1,2], Hyoung Ja Kim[1], Hyungi Chae[1], Na Eun Yoon[1], and Byung Hwa Jung[1,3,*]

HMG-CoA reductase inhibition assay. The crude mixture of AG was extracted sequentially with methanol, dichloromethane, ethyl acetate, and butanol. Among the series of DCQA compounds, 3,5-dicaffeoylquinic acid methyl ester **(3)** showed the most potent activity against HMGR inhibition with an IC_{50} value of 9 μM. An analog of (**2**), 4,5-dicaffeoylquinic acid methyl ester **(4)** also showed a reasonable inhibitory activity of 63 μM (Figure S1).

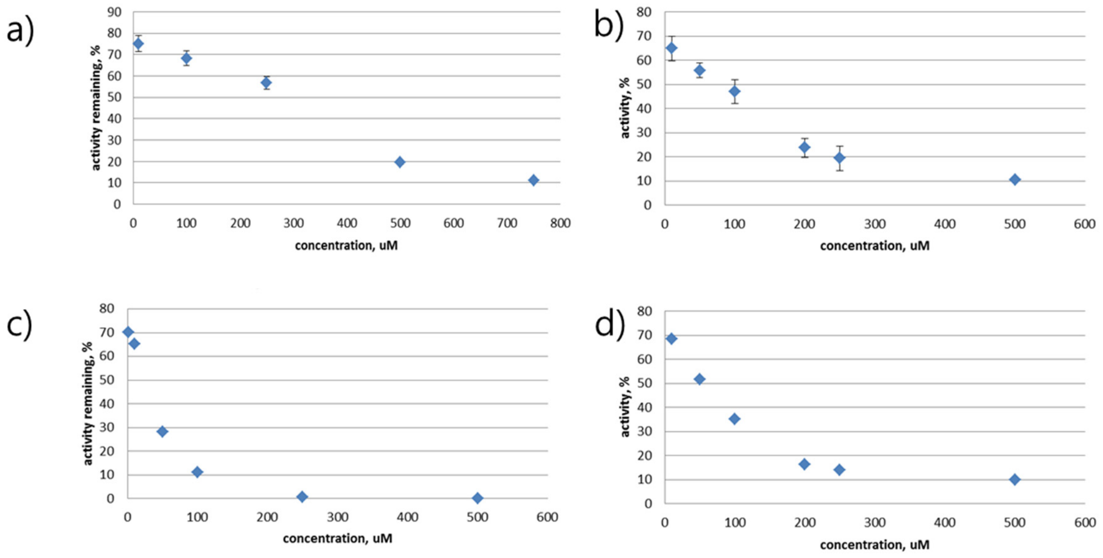

Figure S1. Concentration dependent inhibition of HMGR by single DCQA components isolated from AG. The IC_{50} values were found to be (a)**1 =** 284μM, (b)**2** = 82μM, (c)**3 =** 9μM, and (d)**4 =** 64μM.

Concentration dependent HMG-CoA reductase inhibition assay. The most active fractions, AGEM70M and AGEM90M had IC_{50} values of 19.7±1.4 μg/mL and 18.6±0.85 μg/mL, respectively, against HMGR inhibition (Figure S2).

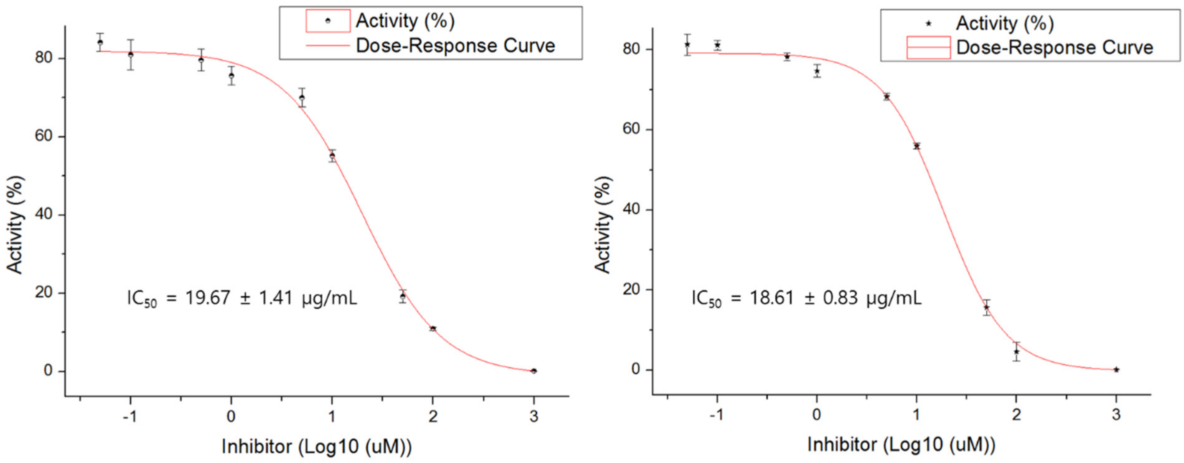

Figure S2. A comparison of the dose-response activity curve for AGEM70M (left) and AGEM90M (right) against HMGR.

Fatty Acid Synthase inhibition assay. Fatty acid synthase activity was tested for a concentration-dependent inhibition with AGEM70M and it showed that it has an IC_{50} value of 106.1 μg/mL (Figure S3).

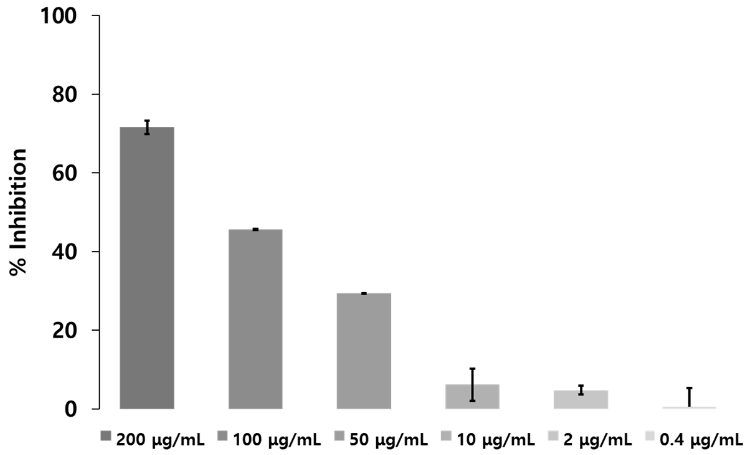

Figure S3. Concentration dependent FAS inhibition assay of the fraction AGEM70M.

Qualitative analysis of components in AGEM. Major peaks were found from the AGEM50M, AGEM70M, and AGEM90M fractions and were identified as the DCQA series from AGEM50M-1, the glucopyranoside series from AGEM50M-2, methylated quercetin, kaempferol, the methylated DCQA series from AGEM70M, and methylated quercetin from AGEM90M (Figure S4). Figure S5 shows the inhibitory activities of the fractions of AGEM70M.

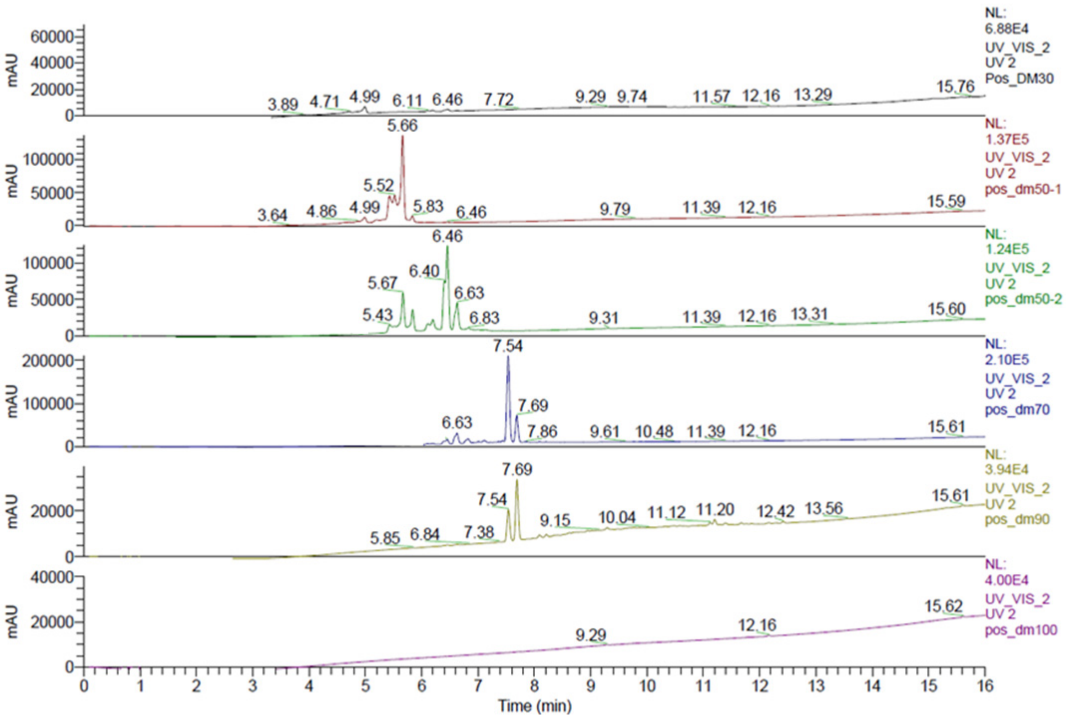

Figure S4. LC-MS/MS chromatogram of AGEM fractions.

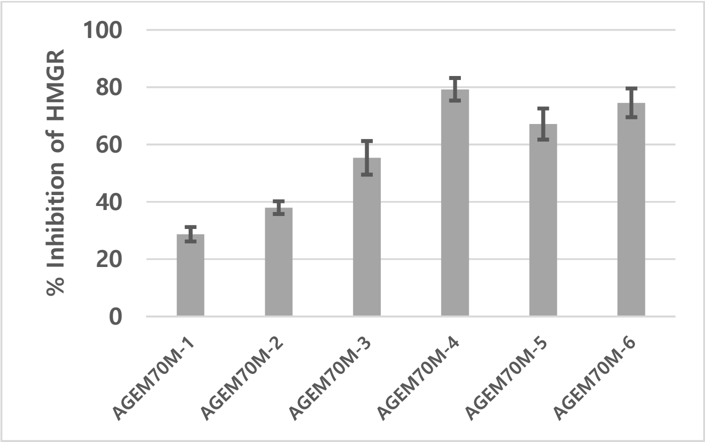

Figure S5. The HMGR inhibitory activity of the fractions (25 μg/mL) of the AGEM70M.

Quantitative analysis of DCQA and isoflavonoids in AGEM fractions. Amount of the contents found in the AGEM70 fractions are listed in the table S1 in nanomolar concentrations.

Table S1. Quantitative analysis of the AGEM70M fractions.

Fraction # (1 ppm)	Kaempferol	3,5-DCQA	4,5-DCQA	3,5-DCQA-ME	4,5-DCQA-ME	Isorhamnetin	Quercetin
**LOQ**	**20**	**1**	**1**	**2**	**5**	**2**	**2**
**AGEM70-1**	< 20	1.85	< 1	< 2	< 5	< 2	< 2
**AGEM70-2**	< 20	9.58	7.49	146	245	< 2	< 2
**AGEM70-3**	< 20	10.4	16.2	142	399	< 2	< 2
**AGEM70-4**	567	5.92	12.9	56.8	223	53.6	3.06
**AGEM70-5**	1450	1.08	1.12	< 2	< 5	98.7	29
**AGEM70-6**	1320	< 1	< 1	< 2	< 5	133	130

(Unit: nM)

Inhibition of HMGR by polyphenols. Polyphenols present in the AG mixtures are mainly found to be kaempferol and quercetin. Since we have performed methylation of the AG fraction, we have tested kaempferol, quercetin, methylated kaempferol (Kaempferol_ME), and methylated quercetin (Quercetin_ME) against HMGR. As expected, polyphenols in the AGEM showed weak HMGR inhibitory effects in a concentration dependent manner. Among the polyphenols, methylated quercetin showed the most potency (Figure S6).

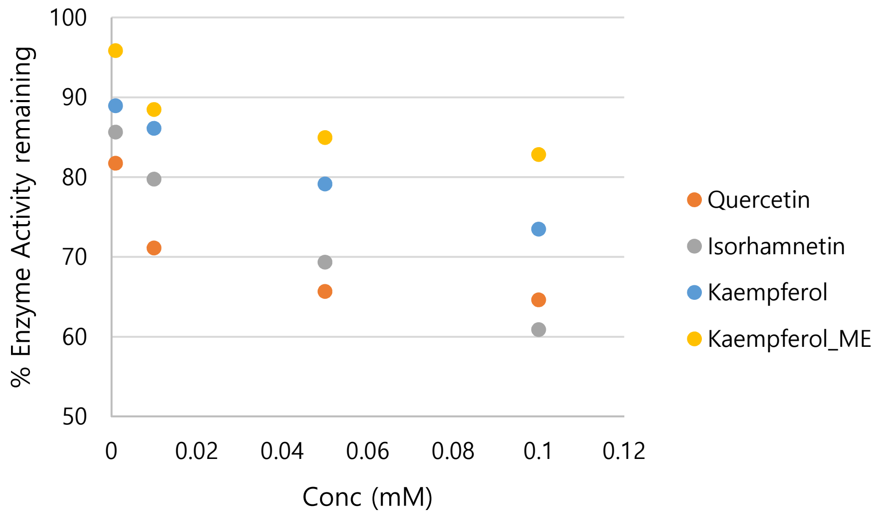

Figure S6. HMGR inhibition by polyphenols present in AGEM.
